# A predictive model to differentiate dengue from other febrile illness

**DOI:** 10.1186/s12879-016-2024-y

**Published:** 2016-11-22

**Authors:** Eduardo Fernández, Marek Smieja, Stephen D. Walter, Mark Loeb

**Affiliations:** 1 Department of Clinical Epidemiology and Biostatistics, McMaster University, Hamilton, Ontario Canada; 2 Department of Pathology and Molecular Medicine, McMaster University, Hamilton, Ontario Canada; 3 Institute for Infectious Diseases Research, McMaster University, Hamilton, Ontario Canada

**Keywords:** Dengue, Fever, Predictive model, Symptoms, Honduras

## Abstract

**Background:**

Dengue is a major public health problem in tropical and subtropical countries and has a presentation similar to other febrile illnesses. Since laboratory confirmation is frequently delayed, the majority of dengue cases are diagnosed based on symptoms. The objective of this study was to identify clinical, hematological and demographical parameters that could be used as predictors of dengue fever among patients with febrile illness.

**Methods:**

We conducted a retrospective cohort study of 548 patients presenting with febrile syndrome to the largest public hospitals in Honduras. Patients’ clinical, laboratory, and demographic data as well as dengue laboratory detection by either serology or viral isolation were used to build a predictive statistical model to identify dengue cases.

**Results:**

Of 548 patients, 390 were confirmed with dengue infection while 158 had negative results. Univariable analysis revealed seven variables associated with dengue: male sex, petechiae, skin rash, myalgia, retro-ocular pain, positive tourniquet test, and gingival bleeding. In multivariable logistic regression analysis, retro-ocular pain petechiae and gingival bleeding were associated with increased risk, while epistaxis and paleness of skin were associated with reduced risk of dengue. Using a value of 0.6 (i.e., 60% probability for a case to be positive based on the equation values), our model had a sensitivity of 86.2%, a specificity of 27.2%, and an overall accuracy of 69.2%; allowing for the diagnosis of dengue to be ruled out and for other febrile conditions to be investigated.

**Conclusions:**

Among Honduran patients presenting with febrile illness, our analysis identified key symptoms associated with dengue fever, however the overall accuracy of our model was still low and specificity remains a concern. Our model requires validation in other populations with a similar pattern of dengue transmission.

## Background

Dengue is a vector-borne flavivirus infection transmitted by *Aedes* mosquitoes that poses a major public health threat in many tropical and subtropical regions [1–3]. Worldwide, dengue affects 50 million people annually and causes 20,000 to 25,000 deaths [1]. In Honduras, 66,814 cases were reported in 2010, with a case fatality rate of 3% [4]. Dengue cases in Honduras are concentrated in urban centers where the affected populations include both school age children and adults. In recent years the incidence has peaked between June and November, with epidemics observed every 24 to 36 months [4].

Clinical manifestations of dengue range from febrile illness to severe complications resulting in hospitalization and death. In its early stages, dengue resembles other febrile illness such as malaria, influenza, and leptospirosis, constituting a diagnostic challenge for clinicians in endemic countries [5]. Further complications to dengue diagnosis arise from the presence of overlapping manifestations such as the presence of hematuria, which can occur in cases of either falciparum malaria or dengue [6, 7]. The ability to differentiate between dengue and other conditions is of prime importance to clinicians in dengue endemic areas. An early diagnosis allows for the early monitoring of indicators that are associated with eventual severe disease.

A confirmatory diagnosis of dengue is made through laboratory diagnosis using serology (e.g., IgM titres, IgG seroconversion), antigen detection of non-structural protein (NS) in sera, or polymerase chain reaction (PCR) for dengue virus nucleic acids in blood. Of these laboratory methods, PCR is not available in many middle and low income countries. Similarly, antigen detection of NS is frequently unavailable or is not part of routine dengue confirmation. Although serology is available in many settings, there may be delays in obtaining results, and, in many areas, no laboratory testing is available at all.

Although there is no specific therapy, identifying dengue can help clinicians make better decisions; for example they can triage patients by knowing which patients need to be monitored for possible dengue complications. On the other hand, dengue diagnosis based solely on clinical presentation is challenging and can lead to misdiagnosis with the ensuing consequences for patients. In Honduras, for example, studies show that less than 50% of specimens tested in cases of suspected dengue are confirmed to be positive, with the remainder being negative or inconclusive [8, 9].

Previous reports exist of models for dengue, using symptoms, signs, and laboratory data to differentiate dengue from other febrile syndromes [10–16]. Demographic factors such as age and sex [12, 13]; clinical symptoms such as joint pain, vomiting, and myalgia [15]; and laboratory findings such leucopenia and thrombocytopenia [16], have all been reported as predictors for dengue. Limitations of these studies include a small sample size [11–14], lack of validation of the models [17], limited geographic representation [14, 15], and limited predictive ability of the results [17–19].

We conducted analyses using data that was systematically collected on a cohort of febrile patients seen at different health care facilities in Honduras. Using these data, we built and validated logistic regression models to identify clinical factors to predict laboratory confirmed dengue.

## Methods

Data on signs and symptoms of patients who present with dengue is routinely collected in Honduras and submitted to the Honduran National Classification Committee for classification of cases. We used such data collected between June 2009 and June 2010 from patients who presented with fever and ≥ 2 symptoms to one of five hospitals and outpatient clinics located in Tegucigalpa and San Pedro Sula, the two largest cities in Honduras. These symptoms included headache, retro-ocular pain, myalgia, rash, anorexia and vomiting, any bleeding manifestation, including petechiae, ecchymosis, epistaxis, gingival bleeding, hematuria, heavy menstrual bleeding, and symptoms suggestive of capillary leakage, including abdominal pain, cold extremities, and sweating. Patients of all ages were included in our analyses, all were tested for dengue and all, if not hospitalized at presentation, were subsequently hospitalized. Patients were seen either at healthcare facilities that are part of the social security system (Instituto Hondureño de Seguridad Social) or not facilities that form part of the public health system. The former serves the insured employed population whereas the latter serves the general population with no restrictions.

General practitioners, nurses, and microbiologists at either the primary health care centres or at the tertiary hospitals completed a data collection form (for case classification) which included epidemiological and clinical information. Symptoms and signs were collected at first presentation while hematology and biochemical tests recorded were those at presentation and those repeated approximately one week later. Fever was defined as a temperature of > 37.5 degrees Celsius. Both objective measurements of fever and self-report of fever were included. Conditions with a febrile presentation, such as malaria, kidney infections, chronic hepatitis, leptospirosis, and respiratory infections were evaluated however complete information on the ultimate diagnosis for patients that tested negative for dengue was either not available or not recorded in the data collection form.

Blood samples were collected from patients for dengue laboratory confirmation. If a patient presented within 5 days of the first symptom onset, samples were processed for viral culture following guidelines from the U.S Centers for Disease Control and Prevention (CDC) [20]. If the blood sample was taken on the sixth day or later of onset of symptoms, a test for antibody detection was done using IgM antibody-capture enzyme-linked immunosorbent (IgM Mac ELISA) from Standard Diagnostic of Bio Venture Company (Gyeonggi-do, South Korea) [20, 21]. This commercial kit reports a sensitivity of 96.4% and specificity of 98.9% [21]. Serology and viral isolation were performed at the Honduras National Laboratory of Virology according to CDC guidelines and standardized techniques.

For hospitalized patients, our analysis was based on serial blood tests taken over the 3 days following admission, and using additional radiological or laboratory testing if available. The initial clinical diagnosis of the treating physician prior to laboratory confirmation was recorded and submitted to the Honduran National Classification Committee for classification as uncomplicated dengue or severe dengue i.e. dengue haemorrhagic fever or shock syndrome [8, 22].

All patients received a laboratory result for dengue (either positive or negative based on viral isolation or IgM). Complete information was obtained for 548 patients. Of 548 patients studied, 390 had laboratory evidence for dengue infection while 158 had negative results.

### Predictor variables

We used the data collected at presentation to assess predictor variables for dengue by grouping these into three categories: 1) general symptoms, including fever, headache, retro-ocular pain, myalgia, rash, anorexia and vomiting; 2) bleeding manifestations, including petechiae, ecchymosis, hematemesis, melena, positive tourniquet, epistaxis (nose bleeding), gingival bleeding, hematuria, and heavy menstrual bleeding (metrorrhagia); and 3) symptoms and signs suggestive of capillary leakage, including abdominal pain, cold extremities, sweating, paleness of skin, serous membranes’ effusion (pericardial, pleural effusion and ascites), and reduction of mean arterial pressure. Hematological results (leucocytes count, and hemoglobin and hematocrit values) were recorded if they were available for at least two consecutive days.

### Power calculation

A sample size was not determined *a priori* since the study enrolled all patients admitted during the study period who had febrile illness and laboratory testing for dengue. Since petechiae are both readily identifiable and are commonly seen in dengue infection we based the study’s power calculation on the predictive value of petechiae to differentiate dengue from other febrile illness [23]. Based on the 548 records (390 dengue-positive and 158 dengue-negative), assuming a prevalence of petechiae of 25% in non-dengue febrile illness and an alpha level of 0.05, the study had 80% power to detect a 1.5-fold increase risk of dengue given the presence of petechiae.

### Data analysis

The chi-square test was used to detect significant differences between presenting signs and symptoms (recorded during admission and on the first day of the patient’s stay, prior to being tested for dengue) and the laboratory tests results.

A logistic regression model was built using a forward step-wise selection. Variables with *p* < 0.2 were entered into the multivariable models. We decided *a priori* to include both age and sex in the analysis. Correlation among pairs of variables was assessed calculating values for tolerance and the variance inflation factor (VIF). Values for tolerance >0.2 and for VIF < 5 were considered as being compatible with a low collinearity. Variables that were statistically significant (*p* < 0.05) were kept in the final model.

Confidence intervals for the odds ratios, were calculated for each predictor. As part of the models’ internal validation, the bootstrap method (i.e. resampling data) was used to estimate the accuracy of the estimators (standard error, confidence intervals and bias) [24–26]. The model was also validated using subsamples, each corresponding to one tenth of the sample and with nine tenths of the remaining sample used for a cross-validation. The subsamples were drawn using systematic sampling (including every tenth case for each subsample) without leaving any patient out of the procedure.

Additionally, as a sensitivity analysis, we created a dichotomous variable for the type of health care facility where patients had presented to either the social security system (accessible only to the insured employed population) or at public health clinics (open to the general population). This was done to account for potential centre effect, that is, differences between the two patient populations.

After building and validating logistic regression models, the sensitivity of the method was determined using the receiver operating characteristic (ROC) curve, which is a graphic plot illustrating the performance of a binary classifier system like the one used during this study.

Using the model equation, we then created a score from the equation by transforming the values assigned to each predictor to integers by multiplying by ten (avoiding decimal values). All statistical analysis were done using SPSS version 19 (IBM) [27].

## Results

The final study sample consisted of 548 patients: 227 were female (41%); mean age 21 years of age (SD = 15.5 years). Of these patients, 295 (53.8%) were admitted to tertiary-level hospitals, 240 (43.8%) in hospitals providing care for insured workers, and 13 (2.4%) originally seen in regional and local health clinics and later admitted to hospital.

Complete clinical and demographic data was available for all 548 patients. However, information on the date of onset of symptoms was missing in 10% of the sample. The duration of signs or symptoms from date of onset is summarized in Table 1. For 495 patients for whom the date of onset of symptoms was available, the mean time for obtaining the dengue sample from onset of symptoms was 6 days (range 0–65 days).

**Table 1:** Duration of signs and symptoms from date of onset of study patients

	0–3 days	4–6 days	≥7 days
	N (%)	N (%)	N(%)
Fever	54(100)	262(98.5)	172(98.3)
Headache	47(87.0)	229(86.1)	147(84.2)
Retroocular Pain	39(72.2)	200(75.2)	125(71.4)
Myalgias	46(85.2)	222(83.5)	151(86.3)
Arthralgias	40(74.1)	193(72.6)	136(77.7)
Skin rash	19(35.2)	92(34.6)	59(33.7)
Anorexia	38(70.4)	218(82.0)	142(81.1)
Petechiae	7(13.0)	73(27.4)	61(34.9)
Epistaxis	9(16.7)	53(19.9)	32(18.3)
Echymoses	1(1.9)	21(7.9)	21(12.0)
Gingival bleeding	2(3.7)	14(5.3)	9(5.1)
Pallor	25(46.3)	123(46.2)	86(49.1)

Viral isolation by cell culture was done in 114 patients who presented within the first 5 days of symptoms, resulting in 9 (7.9%) positive isolates. Serology was done in the remaining 434 patients, 381 (87.8%) of whom were seropositive. Altogether, dengue laboratory testing revealed that 390 patients (71%) were positive (381 by serology and 9 by viral isolation).

The social security system submitted 239 blood samples and 203 (84.9%) tested positive. The public health system submitted 309 samples and 187 (60.5%) tested positive. In terms of type of test requested, viral isolation was requested in 14 patients by the social security system and in 100 patients by the public health system, for a positivity of 28.6% (five cases) and 5% (five cases) respectively. Antibody determination was requested for 225 patients by the social security system and for 209 patients by the public health system, for a positivity of 88.4% (199 patients) and 87.1% (182 patients), respectively.

The mean hematocrit was 40.6 with a range from 17.4 to 40.6 while the mean platelet count at admission was 74,311 with a range from 113 to 61,600. The mean leukocyte count was 5066 (range 513 to 36,000).

Nine variables (petechiae, skin rash, myalgias, retro-ocular pain, tourniquet test, cold limbs, gingival bleeding, epistaxis, and skin paleness) had a *p* value < 0.2 and were thus considered for multivariable analysis (Table 2).

**Table 2:** Variables associated with laboratory confirmed dengue among 548 febrile patients in univariable analysis

Variable	Positive Dengue No (%)	Odds ratio (95% CI)	*P* value
Male	168 (43.5)	1.28 (0.9 to 1.8)	0.20
Petechiae	120 (81.1%)	1.52 (1.0 to 2.3)	0.002
Skin rash	138 (76.7)	1.51 (1.0 to 2.3)	0.046
Myalgia	336 (72.6)	1.52 (0.93 to 2.47)	0.09
Retroocular pain	296 (74.6)	1.51 (1.0 to 2.3)	0.04
Positive Tourniquet test	26 (86.7)	2.75 (0.94 to 8.0)	0.05
Cold limbs	139 (66.8)	0.71 (0.5 to 1.0)	0.08
Gingival bleeding	24 (88.9)	3.4 (1.0 t 11.4)	0.04
Epistaxis	68 (63.6)	0.64 (0.41 to 1.01)	0.06
Paleness	174 (65.9)	0.61 (0.4 to 0.9)	0.009

The following variables were independently associated with dengue in multivariable analysis: petechiae (OR 2.0, 95% CI: 1.3, 3.3), retro-ocular pain (OR 1.7, 95% CI: 1.1, 2.5), gingival bleeding (OR 3.7, 95% CI: 1.1, 13), epistaxis (OR 0.6, 95% CI: 0.4, 1.0), and skin paleness (OR 0.6, 95% CI: 0.4, 0.9) (Table 3). Statistics for collinearity were also applied and the values for tolerance were >0.7 and the VIF (variance inflation factor) was ≤1.3, which represent no significant collinearity among variables tested in the model.

**Table 3:** Final logistic regression model for predictors of dengue

Variable	Odds ratio (95% CI)	*P* value
Petechiae	2.0 (1.3 to 3.3)	0.003
Retroocular pain	1.7 (1.1 to 2.5)	0.01
Gingival bleeding	3.7 (1.1 to 13.0)	0.04
Epistaxis	0.6 (0.4 to 1.0)	0.045
Paleness	0.6 (0.4 to 0.9)	0.006

The model had a sensitivity of 86.2% and a specificity of 27%, with a positive predictive value of 74.5% and a negative predictive value of 44.3%. The positive and negative likelihood ratios were 1.2 and 0.5, respectively, for an overall model accuracy of 69.2%. For the classification of cases (positive cases), the software’s default threshold or cut-point was 0.5; however the specificity was extremely low (4.4%). Therefore, we increased the cut-point to 0.6 which reduced the overall model prediction (from 71.9 to 69.2%) but increased the specificity. In this cases the cut-point defined the level of acceptance of the cases as positive dengue, ROC was used to identify the best cut-point for the classification that provided a higher specificity while keeping a level of sensitivity higher than 85%.

Applying the bootstrap method, we found that *p* values from the subsamples were similar to those obtained in the entire sample, and the bias values were < 25% of the standard error values of the predictors of the subsamples.

Including the health care facility as a variable in the multivariable analysis, as a sensitivity analysis, yielded a model that included two clinical predictors with a positive association to confirmed dengue: petechiae (OR 1.80; 95% CI: 1.11 to 2.92) and gingival bleeding (OR 3.24,95% CI: 0.93,11.26). It also revealed paleness of skin as a negative association (OR 0.61; 95% CI: 0.41, 0.90). The health care facility was also a significant predictor for dengue (OR 3.42; 95%CI: 2.23, 5.26). Retroocular pain and epistaxis did not appear in this model and the odds ratios for the clinical predictors in this model had smaller odds ratios. At a cut point of 0.6, this alternative model correctly classified 69.9% of the patients, with a sensitivity of 84.1% and specificity of 34.8%, representing a slight improvement in overall classification and specificity but with a reduction in sensitivity over the previous model based only on clinical signs and symptoms. The model, which included health care facility, was also validated using the bootstrap test (data not shown). (Table 4).

**Table 4:** Logistic regression model including health system

Variable	Odds ratio (95% CI)	*P* value
Petechiae	1.8 (1.1 to 2.9)	0.02
Gingival bleeding	3.2 (0.93 to 11.3)	0.06
Health system	3.4 (2.2 to 5.3)	<0.001
Paleness	0.6 (0.4 to 0.9)	0.01

In the analysis of ROC we found that the area under the curve was 0.663 (asymptotic 95% CI: 0.616, 0.710). The equation for the model was y = 0.694 + 0.718(petechiae) + 0.516 (retro-ocular pain) + .316(gingival bleeding) – 0.474 (epistaxis)- 0.535 (skin paleness). The results given by the ROC curve showed a logistic regression model with an area under the curve (AUC) of 0.65 (95% CI = 0.60, 0.70) and a standard error of 0.025, being statistically significant. The logistic regression model had a good overall prediction and this was confirmed through the ROC analysis.

Using the equation, a score was obtained by rounding up values of the equation to the close integer, with the next values. Therefore, petechiae = 7, retroocular pain = 5, gingival bleeding = 13 (all when the sign is present) and epistaxis = −5 and skin paleness = −5 (negative values for when they are present). When only positive predictors are present, the maximum score would be 25 whereas when only negative predictors are present the score would be −10. Intermediate values can be obtained with different combination of predictors.

The score was simplified using the values of the equation and reducing it to values 0 and 1 assigning 1 to those considered cases and 0 to non-cases. That is, signs and symptoms scores were added and a threshold value of 3 was selected to differentiate cases from non cases, (any value ≤ 3 was coded as 0 and values >3 were coded as 1). This resulted in 336 of the 390 positive correctly predicted, and 43 of the 158 negative patients predicted as no dengue cases. The sensitivity and specificity for this score were 86.2% and of 27%, respectively. The area under the curve was 0.567(asymptotic 95% CI were 0.512, 0.622) with a standard error of 0.028. The accuracy is still low if is intended to operationalize the equation for clinical personnel providing a rapid method to identify cases of dengue.

## Discussion

Our logistic regression model found that five symptoms or signs helped differentiate dengue from other febrile illness: [1] petechiae, [2] retro-ocular pain, [3] gingival bleeding, [4] epistaxis and [5] skin paleness. Of these, the association was positive for the first three (likely to be present in dengue cases), and negative for the last two (epistaxis and pale skin), consistent with being less likely to be present in dengue cases.

Our analysis used similar variables to that of previous reported models, but in contrast to previous reports, we not only built and validated a model but created scoring system to assess the model. A cross-sectional study to differentiate dengue from non-dengue among patients attending the emergency department of a public hospital in Brazil was described in one report (10). Only conjunctival redness and leukocyte count were associated with dengue. The sensitivity and specificity of this model was 80.8 and 71.1% respectively but did not include validation techniques. Ramos et al. used surveillance data from Puerto Rico and reported that rash and either the absence of sore throat, nasal congestion, or cough had low (<50%) sensitivity for ruling out dengue in children (11). However, validation and construction of a predictive rule was not conducted. In another study from Puerto Rica using surveillance data, five variables (retro-orbital pain, rash, low platelet count, lack of sore throat, absence of cough) were used to build models that had AUC of 0.76. However, a cut-off point for diagnostic accuracy was not reported (13). In a study from Papa New Gunia, only facial flushing and male sex were associated with dengue in multivariable analysis (14). The AUC was 0.59 but no cut-off was used to predict cases. In a study from Nicaragua among children, fever, headache, retro-orbital pain, myalgia, arthralgia, rash, petechiae, positive tourniquet test, vomiting, leukopenia, platelets ≤150,000 cells/mL, poor capillary refill, cold extremities and hypotension.were associated wtth dengue. This study did not use the model to predict dengue in a validation set.

Including clinical signs in the model make intuitive sense and we consider it to be a strength. Petechiae and gingival bleeding, for example, represent subcutaneous or mucosal bleeding and are therefore readily noticeable by the patient and reported as such. Epistaxis can be frequently associated with respiratory conditions rather than with dengue, and in our sample most patients with epistaxis had a negative dengue test.

With respect to effect size, gingival bleeding had a higher odds ratio than other predictors but also had a wider confidence interval (OR 3.7, CI: 1.1, 13.0.) followed by petechiae, retro-ocular pain. Compared to other studies, our odds ratios were smaller for petechiae, retro-orbital pain and gingival bleeding but clearly represented a positive association with dengue positivity [10, 23, 28–34].

Unlike other studies that have found epistaxis and skin paleness positively associated with dengue [3, 10, 32, 34], our study identified an inverse association with both. Most of our patients with epistaxis had a negative dengue result. Mittal et al. reported that skin paleness or pallor was associated with advanced stages of dengue. In fact, 13.3% of their patients with dengue were described as having pallor [34].

Paleness of skin has been found associated to dengue presumably because of vascular events (bleeding, vasoconstriction) occurring when dengue evolves into severe forms. In Central America, as in other developing regions, paleness can be associated with chronic anemia and malnutrition but frequently the clinician is faced with limited laboratory resources and must resort to basic clinical evaluation to make a diagnosis.

Notably, proportionally more patients from the social security system hospitals were diagnosed correctly (84.9%) than those from the public health system (60.5%) as described in the results. The most likely explanation is that there were proportionately more patients in the public health system that had specimens sent for viral isolation at later time points (beyond 3 days) and that this resulted in a lower detection rate.

The model that included health care facility reduced the number of symptoms to three: petechiae, gingival bleeding and paleness of skin, keeping the same direction of association and similar effect size as the model without health system. The sensitivity of the model increased slightly to 84.1%, and the specificity to 34.8%, for an overall accuracy of 69.9%. Including the health care facility as a predictor increased the accuracy of the model but resulted in a reduction in the number of clinical predictors. Although this finding may suggest differences in the populations receiving care at different levels of the Honduran health system, it is most probably due to differences in the clinical judgment of treating physicians at those levels.

A strength of this study is that it was conducted during a period of active dengue transmission allowing for a sufficient number of patients to be assessed. One limitation is that our sample may have been characterized by an overrepresentation of symptoms deemed important to a subgroup of patients in the early stages of the syndrome, particularly with respect to pain (e.g. headache, retro-ocular pain). Also, the difference in the clinical diagnostic accuracy between health care facilities providing information is a factor to consider as this may represent different levels of experience with the disease. Another potential limitation is that our study was conducted when other viruses, such as Chikungunya and Zika, were not circulating. There is a possibility that this would have affected the predictive value of our model. Since we conducted an analysis using a database on suspected dengue, we did not have access to the final diagnosis of non-dengue cases.

Prior laboratory data in Honduras shows that around 40% of suspected cases with confirmed to be positive by laboratory testing [8, 9]. An important limitation of our study is that IgM testing was not conducted on all samples on days 4 and 5 [35]. We were also unable to differentiate between primary and secondary dengue and this could have affected results because of the predominance of different symptoms in primary versus secondary dengue. The fact that most patients were admitted after 5 days following symptom onset may limit generalizability. It is possible that our sample may have been biased by spectrum, with a representation of patients with more severe illness including symptoms of more severe disease which is observed in patients consulting the medical service but not in the community where milder disease is more frequent. The retrospective nature of our study is another limitation. To confirm the utility of this model it should be tested both during epidemic and inter-epidemic periods.

## Conclusions

In conclusion, we demonstrate that a simple model may identify useful predictors to differentiate between dengue and other causes of febrile illness. The model should optimally be used with a method for rapid confirmatory testing. The use of such a model can define those cases more likely to be dengue and needing primarily a dengue test while other patients can be directed to a different management. The use of a score table as the one presented in the results can facilitate the effort of those working in the screening of febrile illness requiring hospitalization.

## References

[1] WHO. Dengue and dengue haemorrhagic fever. Geneva: World Health Organization; 2008. (updated March 2014; cited 2008 May 2008]; Factsheet No. 117]. Available from: http://www.who.int/mediacentre/factsheets/fs117/en/.

[2] Gubler DJ. Dengue and dengue hemorrhagic fever: its history and resurgence as a global public health problem. In: Gubler DJK, G., editor. Dengue and dengue hemorrhagic fever. CAB International Press; 1997. p. 1–22.

[3] Halstead SB (1988). Pathogenesis of dengue: challenges to molecular biology. Science.

[4] PAHO (2012). Health in the Americas 2012.

[5] Rodriguez LE, Tomashek KM, Gregory CJ, Munoz J, Hunsperger E, Lorenzi OD, et al. Co-infection with dengue virus and pandemic (H1N1] virus (letter]. 2010, et al. Emerg Infect Dis (serial on the Internet]. 2010 May (date cited]. http://wwwnc.cdc.gov/eid/article/16/5/09-1920 Doi: 10.3201/eid1605.091920. 10.3201/eid1605.091920 PMC2954016 20409395

[6] Wiwanitkit V (2011). Concurrent malaria and dengue infection: a brief summary and comment. Asian Pac J Trop Biomed.

[7] Kaushik RM, Varma A, Kaushik R, Gaur KJ (2007). Concurrent dengue and malaria due to *Plasmodium falciparum* and *P. vivax*. Trans R Soc Trop Med Hyg.

[8] Secretaria de Salud (2005). Boletin epidemiologico semanal.

[9] Mendez J, Fernandez EA (1996). Taller sobre avances recientes en el control del *Aedes aegypti* basado en la comunidad: Honduras y Mexico. Merida, Yucatan.

[10] Daumas RP, Passos SR, Oliveira RV, Nogueira RM, Georg I, Marzochi KB (2013). Clinical and laboratory features that discriminate dengue from other febrile illnesses: a diagnostic accuracy study in Rio de Janeiro, Brazil. BMC Infect Dis.

[11] Ramos MM, Tomashek KM, Arguello DF, Luxemburger C, Quinones L, Lang J (2009). Early clinical features of dengue infection in Puerto Rico. Trans R Soc Trop Med Hyg.

[12] Gregory CJ, Santiago LM, Arguello DF, Hunsperger E, Tomashek KM (2010). Clinical and laboratory features that differentiate dengue from other febrile illnesses in an endemic area--Puerto Rico, 2007–2008. Am J Trop Med Hyg.

[13] Phuong HL, de Vries PJ, Nga TT, Giao PT, Hung le Q, Binh TQ (2006). Dengue as a cause of acute undifferentiated fever in Vietnam. BMC Infect Dis.

[14] Senn N, Luang-Suarkia D, Manong D, Siba PM, McBride WJ (2011). Contribution of dengue fever to the burden of acute febrile illnesses in Papua New Guinea: an age-specific prospective study. Am J Trop Med Hyg.

[15] Biswas HH, Ortega O, Gordon A, Standish K, Balmaseda A, Kuan G (2012). Early clinical features of dengue virus infection in nicaraguan children: a longitudinal analysis. PLoS Negl Trop Dis.

[16] Dietz VJ, Gubler DJ, Rigau-Perez JG, Pinheiro F, Schatzmayr HG, Bailey R (1990). Epidemic dengue 1 in Brazil, 1986: evaluation of a clinically based dengue surveillance system. Am J Epidemiol.

[17] Potts JA, Rothman AL (2008). Clinical and laboratory features that distinguish dengue from other febrile illnesses in endemic populations. Trop Med Int Health (Review].

[18] Karande S, Gandhi D, Kulkarni M, Bharadwaj R, Pol S, Thakare J (2005). Concurrent outbreak of leptospirosis and dengue in Mumbai, India, 2002. J Trop Pediatr.

[19] Deparis X, Murgue B, Roche C, Cassar O, Chungue E (1998). Changing clinical and biological manifestations of dengue during the dengue-2 epidemic in French Polynesia in 1996/97--description and analysis in a prospective study. Trop Med Int Health.

[20] Balmaceda A (2002). Manual de procedimientos de tecnicas para el diagnostico del dengue: Organizacion Panamericana de la Salud, Salud DdDdSdSd.

[21] Kuno G, Gomez I, Gubler DJ (1991). An ELISA procedure for the diagnosis of dengue infections. J Virol Methods.

[22] Dirección de Vigilancia de la Salud (2003). Lineamientos de Vigilancia y Manejo Estandarizado de Pacientes con dengue.

[23] Kalayanarooj S, Vaughn DW, Nimmannitya S, Green S, Suntayakorn S, Kunentrasai N (1997). Early clinical and laboratory indicators of acute dengue illness. J Infect Dis.

[24] Schlesselman JJ (1982). Case–control Studies: design, conduct, analysis.

[25] Hesterberg T, Moore DS, Monaghan S, Clipson A, Epstein R, Moore DS, McCabe GP (2005). Bootstrap Methods and Permutation tests. Introduction to the Practice of Statistics.

[26] Efron B (1979). Bootstrap Methods: Another look at the Jacknife. Ann Stat.

[27] SPSS I (2010). SPSS for Windows (version 19].

[28] Phuong CX, Nhan NT, Kneen R, Thuy PT, van Thien C, Nga NT (2004). Clinical diagnosis and assessment of severity of confirmed dengue infections in Vietnamese children: is the world health organization classification system helpful?. Am J Trop Med Hyg.

[29] Chadwick D, Arch B, Wilder-Smith A, Paton N (2006). Distinguishing dengue fever from other infections on the basis of simple clinical and laboratory features: application of logistic regression analysis. J Clin Virol.

[30] Sawasdivorn S, Vibulvattanakit S, Sasavatpakdee M, Iarnsirithavorn S (2001). Efficacy of clinica diagnosis of dengue fever in paediatric age groups as determined by WHO case definition 1997 in Thailand. Dengue Bull.

[31] Nunes-Araujo FR, Ferreira MS, Nishioka SD (2003). Dengue fever in Brazilian adults and children: assessment of clinical findings and their validity for diagnosis. Ann Trop Med Parasitol.

[32] Hammond SN, Balmaseda A, Perez L, Tellez Y, Saborio SI, Mercado JC (2005). Differences in dengue severity in infants, children, and adults in a 3-year hospital-based study in Nicaragua. Am J Trop Med Hyg.

[33] Diaz-Quijano FA, Martinez-Vega RA, Villar-Centeno LA (2005). Early indicators of severity in dengue virus infection. Enferm Infecc Microbiol Clin.

[34] Mittal H, Faridi MM, Arora SK, Patil R (2012). Clinicohematological profile and platelet trends in children with dengue during 2010 epidemic in north India. Indian J Pediatr.

[35] Mishra B, Gupta PK, Dhiman V, Pujhari SK, Sharma M, Ratho RK (2014). Clinical Applicability of Various Dengue Diagnostic Tests in Resource-Limited Endemic Settings. J Global Infect Dis.

